# MicroRNA-497 impairs the growth of chemoresistant neuroblastoma cells by targeting cell cycle, survival and vascular permeability genes

**DOI:** 10.18632/oncotarget.7005

**Published:** 2016-01-25

**Authors:** Aroa Soriano, Laia París-Coderch, Luz Jubierre, Alba Martínez, Xiangyu Zhou, Olga Piskareva, Isabella Bray, Isaac Vidal, Ana Almazán-Moga, Carla Molist, Josep Roma, José R. Bayascas, Oriol Casanovas, Raymond L. Stallings, José Sánchez de Toledo, Soledad Gallego, Miguel F. Segura

**Affiliations:** ^1^ Laboratory of Translational Research in Child and Adolescent Cancer, Vall d'Hebron Research Institute (VHIR)-UAB, Barcelona, Spain; ^2^ Tumor Angiogenesis Group, Catalan Institute of Oncology-IDIBELL, L'Hospitalet de Llobregat, Barcelona, Spain; ^3^ Institut de Neurociències and Departament de Bioquímica i Biologia Molecular, Universitat Autònoma de Barcelona, Barcelona, Spain; ^4^ Molecular and Cellular Therapeutics, Royal College of Surgeons in Ireland and National Children's Research Centre Our Lady's Children's Hospital, Dublin, Ireland

**Keywords:** microRNA, neuroblastoma, epigenetic therapy, vascular permeability

## Abstract

Despite multimodal therapies, a high percentage of high-risk neuroblastoma (NB) become refractory to current treatments, most of which interfere with cell cycle and DNA synthesis or function, activating the DNA damage response (DDR). In cancer, this process is frequently altered by deregulated expression or function of several genes which contribute to multidrug resistance (MDR). MicroRNAs are outstanding candidates for therapy since a single microRNA can modulate the expression of multiple genes of the same or different pathways, thus hindering the development of resistance mechanisms by the tumor. We found several genes implicated in the MDR to be overexpressed in high-risk NB which could be targeted by microRNAs simultaneously. Our functional screening identified several of those microRNAs that reduced proliferation of chemoresistant NB cell lines, the best of which was miR-497. Low expression of miR-497 correlated with poor patient outcome. The overexpression of miR-497 reduced the proliferation of multiple chemoresistant NB cell lines and induced apoptosis in MYCN-amplified cell lines. Moreover, the conditional expression of miR-497 in NB xenografts reduced tumor growth and inhibited vascular permeabilization. MiR-497 targets multiple genes related to the DDR, cell cycle, survival and angiogenesis, which renders this molecule a promising candidate for NB therapy.

## INTRODUCTION

Neuroblastoma (NB) is an extracranial neoplasm originating in the neural crest lineage of the sympathetic nervous system. It is considered an embryonal cancer [1] and occurs mainly in children between 0 and 4 years of age. NB has an incidence in Europe and North-America of 10.5 cases per million in children and adolescents and accounts for 12 to 15% of all cancer-related deaths in children [2, 3]. NB patients are assigned to three different risk categories according to clinicopathologic variables such as age at diagnosis, MYCN oncogene amplification, tumor histology and DNA ploidy. Overall survival for low-risk patients is excellent; minimal intervention is required and, on occasions, surgery alone suffices. Intermediate-risk patients generally have good prognosis and are treated with surgery and standard chemotherapy. However, high-risk patients have poor prognosis and need intense chemotherapeutic regimens. Despite the aggressive treatment, 50–60% of these patients will not achieve long-term cure owing to disease progression and resistance to current therapies [4–7]. Acquired drug resistance is often characterized by multiple drug resistance (MDR), which is defined as insensitivity of cancer cells to cytotoxic and cytostatic action of a number of structurally and functionally unrelated chemotherapeutic agents [8]. In general, this MDR is a major obstacle to the success of chemotherapy. Some key elements have been shown to participate in the MDR process in NB, and include the increased expression of oncogenes such as MYCN, TrkB/BDNF signaling, or alterations in the DNA damage response (DDR) elements such as p53 [9–12], CHEK1 [13] or PLK1 [14, 15], among others. It has also been demonstrated that major elements of the apoptotic signaling cascade, including BCL2 family members, survivin and caspase-8 present abnormal expression or activation patterns [16, 17]. In addition, acquired resistance to chemotherapeutic agents may be produced by enhanced drug efflux due to overexpression of membrane transporters [18, 19].

Owing to the multiple mechanisms that lead to NB resistance to therapy, targeting single elements of a pathway may not suffice. In this respect, it is desirable to find molecules, such as microRNAs, that can regulate multiple cellular processes, thereby minimizing the risk of resistance and improving the clinical response. MicroRNAs (miRNAs) are endogenous small non-coding RNAs that regulate gene expression by direct binding to the 3′untranslated (UTR) region of mRNAs, blocking their translation and/or inducing their degradation [20]. MiRNAs are upstream regulators that can simultaneously target large numbers of protein-coding genes and multiple cancer pathways. Emerging cumulative data reveal functional roles of miRNAs in the origin and progression of NB [21]. Recently, an overall reduction in miRNAs was observed in advanced NB, mainly due to alterations in the miRNA processing machinery [22]. Therefore, miRNA(s) restoration represents an attractive novel therapeutic approach against aggressive tumors that do not respond to conventional chemotherapies.

In order to select the best miRNA candidates, we sought to analyze the expression levels of the MDR-related genes in human NB samples and select those that were highly expressed in the most aggressive NBs (Stage 4, MYCN-amplified tumors). 3′UTR analysis of those genes revealed that most of them contain binding sites for various miRNAs in their sequence. Those miRNAs that were capable of targeting 3 or more MDR-altered genes simultaneously were then selected for functional analysis in cellular and animal models of chemoresistant NB. Our strategy permitted the identification of miR-497, the restoration of which impairs cellular proliferation *in vitro* and *in vivo*. MiR-497 can be a new therapeutic tool for NB therapy due to its ability to target multiple genes related to cell cycle, cell survival, and angiogenesis regulators.

## RESULTS

### Several DNA-damage response and detoxification-associated genes are overexpressed in advanced neuroblastoma

**Figure 1:**
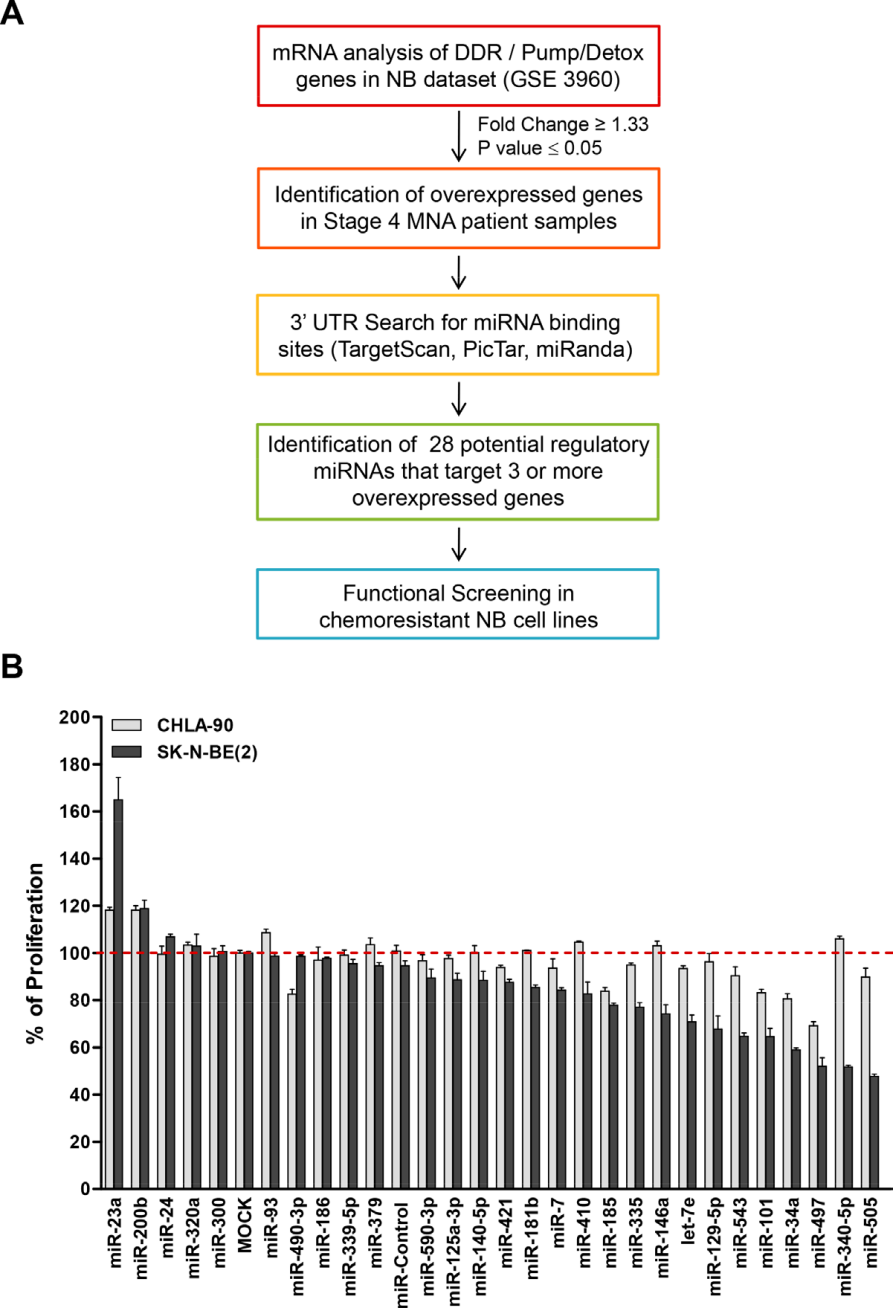
Functional screening of selected miRNAs (**A**) Diagram of *in silico* analysis carried out to select miRNAs candidates. (**B**) Twenty-eight different mimic miRNAs oligonucleotides were reverse-transfected in the chemoresistant NB cell lines CHLA-90 (MYCN non-amplified, gray bars) and SK-N-BE(2) (MYCN amplified, black bars). Cells were fixed and stained with crystal violet 96 h post-transfection. Absorbance was measured at 590 nm after dissolving the crystals with 15% acetic acid. Proliferation values were normalized versus MOCK-transfected cells. Data represent mean ± SEM of three independent experiments (six replicates each experiment). Statistical significance was determined by two-tailed unpaired Student's *t*-test comparing each miRNA versus miR-Control. *P*-values for the CHLA-90 cell line are miR-185: 0.00012, miR-101: 4.55 × 10^−05^, miR-34a: 0.00028 and miR-497: 4.13 × 10^−07^. *P*-values for the SK-N-BE(2) cell line are miR-185: 5.05 × 10^−06^, miR-101: 0.00311, miR-34a: 5.17 × 10^−10^ and miR-497: 0.00105.

The expression of the most important MDR-associated genes (DDR mediators and pumps and detoxification genes) was analyzed in different stages of human NB tumors [23] and whether they could be simultaneously targeted by miRNAs was explored (Figure 1A). Twenty-three out of forty-five genes were found to be overexpressed in the worst prognosis NB group (Stage 4, MYCN amplified) (Supplementary Table 1). Analysis of the 3′UTR of the overexpressed genes showed almost all of them to being susceptible to be regulated by miRNAs. Twenty-eight different miRNAs were found to be potential regulators of at least 3 different analyzed genes (Supplementary Table 2) by at least two independent miRNA-binding site prediction algorithms (i.e. TargetScan [24], PicTar [25] and miRANDA [26]).

### The miR-15 family member miR-497 has the highest therapeutic potential

The effects of miRNA expression listed in Supplementary Table 2 were evaluated in two different NB cell lines derived from tumors resistant to currently-used chemotherapeutic drugs (i.e. cisplatin, etoposide, melphalan) at clinically-achievable doses in children [27]. MiRNAs were reverse-transfected into CHLA-90 and SK-N-BE(2) NB cell lines and proliferation was evaluated at 96 h post-transfection. Figure 1B shows that 4 of 28 miRNAs (miR-185, miR-101, miR-34a and miR-497) did significantly reduce the number of cells of both cell lines compared to MOCK-transfected cells or versus non-targeting miRNA (miR-Control), with miR-497 being the miRNA with the clearest effects in both NB cell lines.

**Figure 2:**
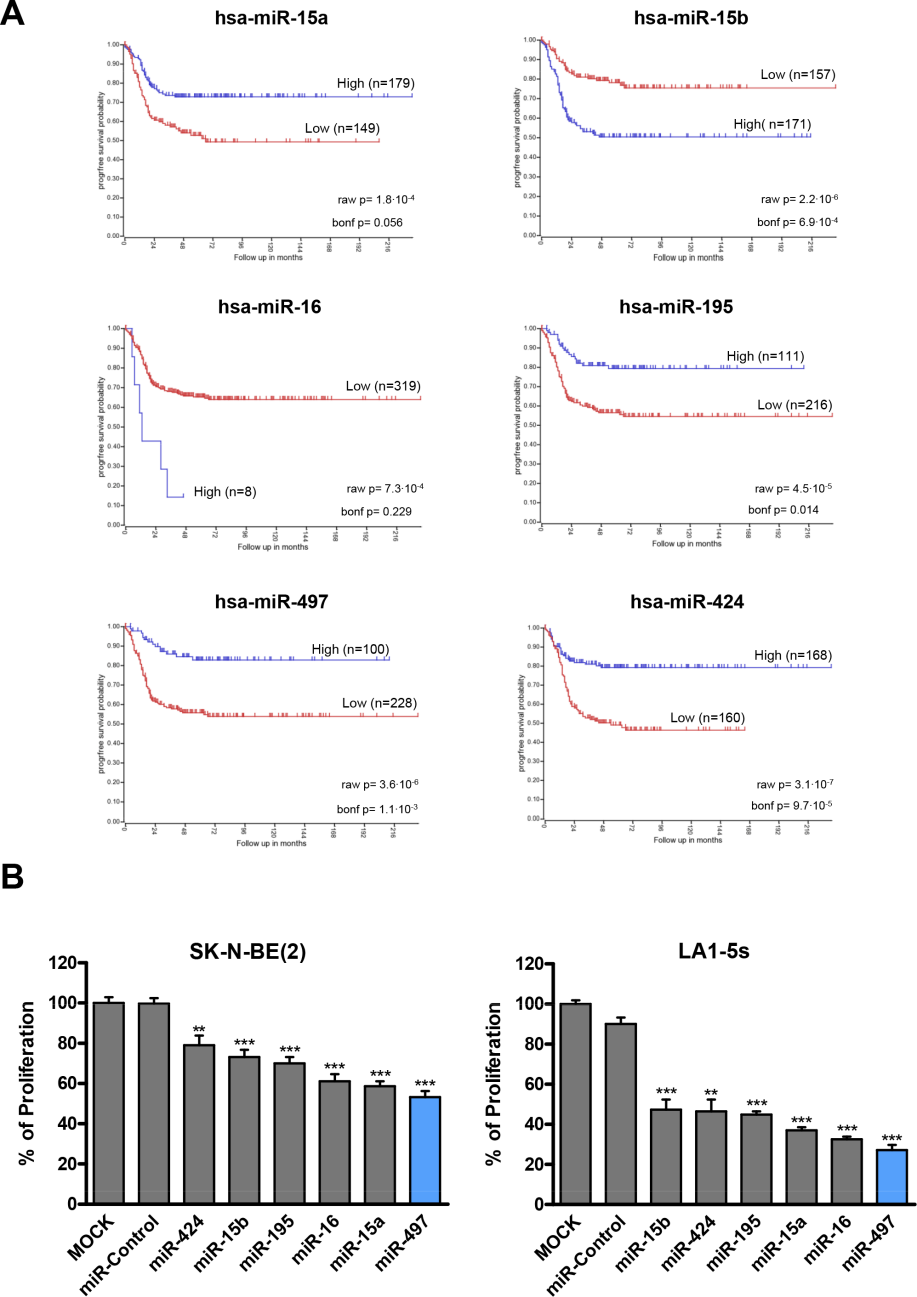
MiR-15 family members expression correlates with NB prognosis and regulates cell proliferation (**A**) Kaplan-Meier progression-free survival analysis of miR-15 family members in human NB tissues (*n* = 328). (**B**) The miR-15 family members (miR-15a, miR-15b, miR-16-1/2, miR-195, miR-497 and miR-424) were reverse-transfected in SK-N-BE(2) and LA1-5s cells. 96 h later, cells were fixed and stained with crystal violet. Proliferation values were normalized versus MOCK-transfected cells. Data represent mean ± SEM of three independent experiments (six replicates per experiment). **p < 0.01; ***p < 0.001.

MiR-497 belongs to the miR-15 family which has five additional members (miR-15a, miR-15b, miR-16-1/2, miR-195 and miR-424); all share the same seed region, and thereby potentially regulate a similar pool of targets (Supplementary Table 3). When the expression of the miR-15 family members was analyzed in human NB tissues (*n* = 328), the low expression of miR-15a, miR-195, miR-497 and miR-424 correlated with worse progression-free survival (Figure 2A). Tumors from patients with MYCN amplification (poor outcome) also showed reduced levels of miR-195, miR-497 and miR-424 (Supplementary Figure 1). These data suggest that replacement of miR-15 family members could be exploited therapeutically. In order to clarify whether the diverse family members could have different therapeutic potential, we compared the effects of transfecting all 6 individual miRNAs on the proliferation of NB cells. MiR-497 was the miR-15 family member which reduced the number of viable NB cells the most (Figure 2B).

### MiR-497 reduces proliferation of chemoresistant NB cells and induces apoptosis in MYCN-amplified cell lines

**Figure 3:**
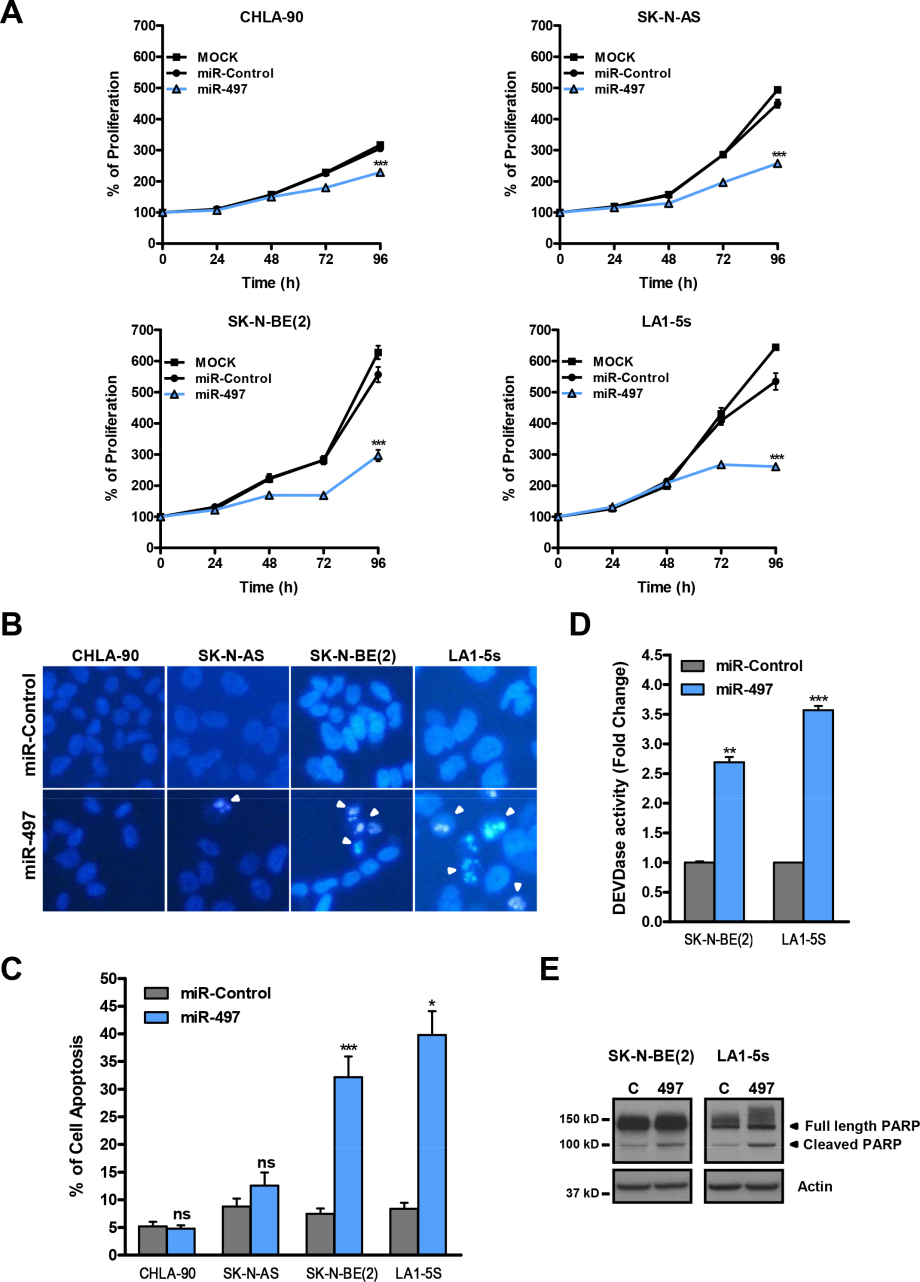
MiR-497 overexpression reduces proliferation of chemoresistant NB cells and induces apoptosis in MYCN-amplified NB cells (**A**) Proliferation time course comparing miR-497 versus miR-Control (25 nM) reverse transfected in CHLA-90 and SK-N-AS cells (both non-MYCN amplified) or SK-N-BE(2) and LA1-5s cells (both MYCN amplified). (**B**) Representative images of nuclear morphology assessment at 96 h post-transfection with Hoechst staining in miR-Control and miR-497 (25 nM) reverse transfected NB cell lines. Arrowheads point at condensed or fragmented nuclei. (**C**) Quantification of apoptosis was performed from 4 representative images of 3 replicates per condition. (**D**) Caspase-3/7 activity assays and (**E**) representative Western blot of PARP protein at 72 h post-transfection. SK-N-BE(2) and LA1-5s cells were reverse transfected with 25 nM of miR-Control or miR-497. Data represent mean ± SEM of three independent experiments *, ** or *** indicated significant differences comparing miR-497 *versus* miR-Control at *p* < 0.05, *p* < 0.01 or *p* < 0.001, respectively.

The effects of miR-497 were then analyzed in a panel of NB cell lines representative of the major subclasses of NB (MYCN-amplified and non-amplified) over a time-course period. A reduction in cell proliferation started to be visible in all cell lines at 72 h post-transfection (Figure 3A).

To further ascertain whether the effects of miR-497 were due to a reduction in cell proliferation and/or increased cell death, the induction of apoptosis was analyzed in miR-497-transfected cells. The number of cells with condensed or fragmented chromatin (one of the hallmarks of apoptotic cell death) was found to be increased upon miR-497 transfection in MYCN-amplified (SK-N-BE(2) and LA1-5s) but not in MYCN-non amplified cell lines (CHLA-90 and SK-N-AS) (Figure 3B–3C). Furthermore, the implication of caspases in miR-497-induced cell death was confirmed with a caspase activity assay (Figure 3D) and the cleavage of caspase-3/7 substrate PARP (Figure 3E).

In summary, our results show that the sole ectopic expression of miR-497 suffices to reduce the proliferation of all tested chemoresistant NB cell lines and induce cell death in MYCN-amplified NB cells.

### MiR-497 overexpression reduces tumor growth and vascular permeability

**Figure 4:**
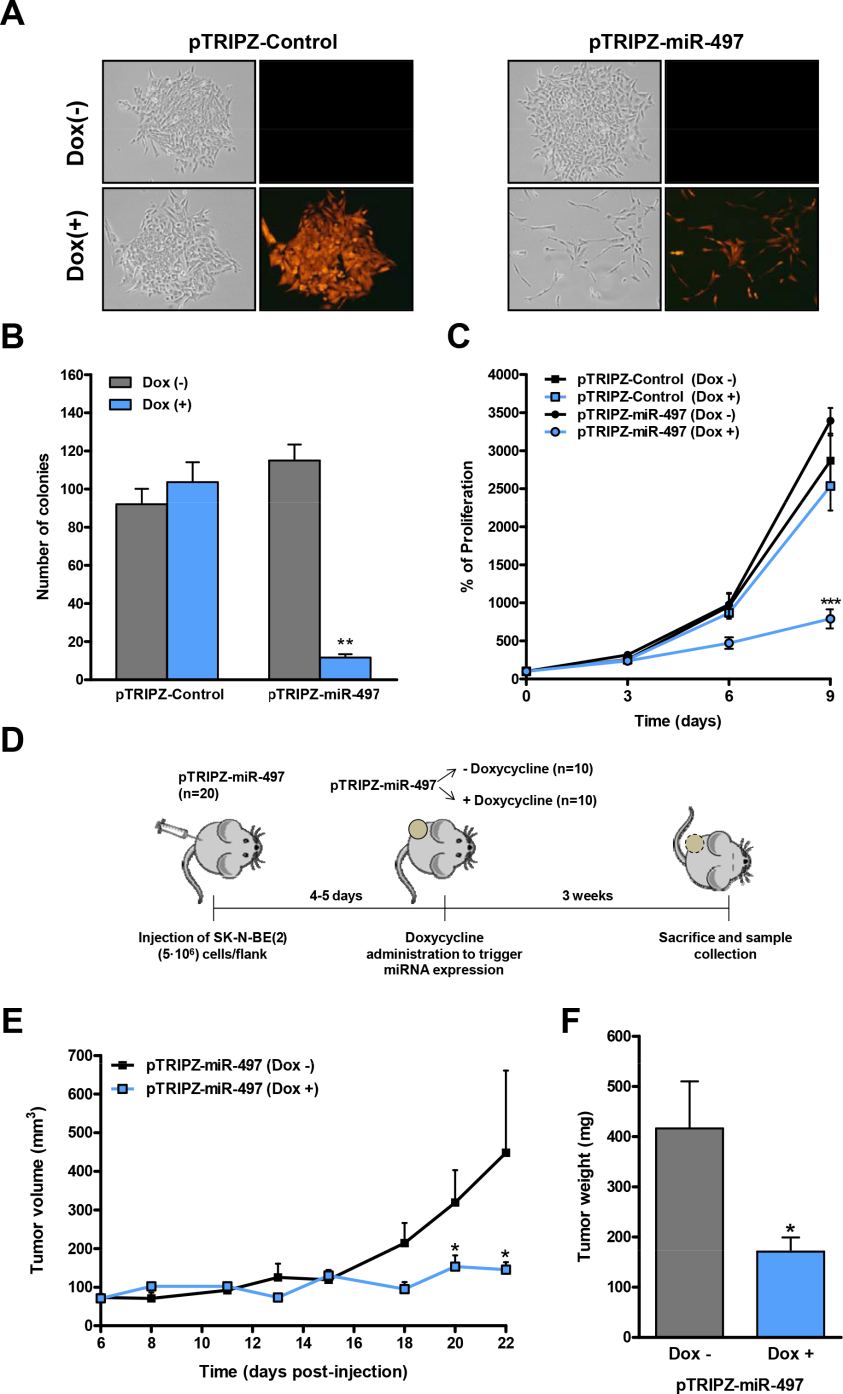
Inducible expression of miR-497 impairs colony formation and proliferation *in vitro* and tumor growth *in vivo* (**A**) Representative images of a colony-formation assay in pTRIPZ-Control or pTRIPZ-miR-497 stable SK-N-BE(2) cells treated with or without doxycycline (0.5 μg/mL) and allowed to grow for 13 days (*n* = 3). Expression of the TurboRFP permits the visual marking of miRNA-expressing cells. (**B**) Colonies were stained with crystal violet, photographed and the number of colonies was quantified. (**C**) Proliferation time course comparing pTRIPZ-Control or pTRIPZ-miR-497 stable SK-N-BE(2) cells with or without 0.5 μg/mL of doxycycline. Data represent mean ± SEM of three independent experiments (3 replicates per condition for each experiment). ** or *** indicated significant difference comparing cells with or without doxycycline at *p* < 0.01 or *p* < 0.001, respectively. (**D**) Mouse model design. 5 · 10^6^ of SK-N-BE(2) stably transduced cells with pTRIPZ-miR-497 (*n* = 20) were injected into the right flank of NMRI-nude mice. Once tumors were detected, mice were randomized in two groups (*n* = 10/group). MiR-497 expression was then induced with the administration of 1 mg/mL doxycycline with 2% sucrose in drinking water *ad libitum*. Tumor measurements were taken every 2–3 days for three weeks. Tumor growth (**E**) and weight (**F**) of NB xenografts derived from SK-N-BE(2)-pTRIPZ-miR497 cells. * means *p* < 0.05.

Since our data pointed to a therapeutic potential of miR-497 against refractory NB, we proceeded to engineer an inducible miR-497 expression system to evaluate the effects of miR-497 expression *in vivo*. The precursor form of miR-497 (pre-miR-497, accession # MI0003138) was cloned into the Tet-On lentiviral inducible vector (pTRIPZ, Thermo) which provides inducible expression of the miRNA in the presence of doxycycline. SK-N-BE(2) cells were transduced with either pTRIPZ-Control or pTRIPZ-miR-497 and stable cells were selected with puromycin. The addition of different concentrations of doxycycline (0.1–1 μg/ml) to the culture media of pTRIPZ-miR-497-transduced cells induced miR-497 overexpression, obtaining the highest overexpression at 0.5 μg/ml (∼40-fold) (Supplementary Figure 2A). The miR-497-expressing cells can be monitored by following the red fluorescent protein (Supplementary Figure 2B). The functional consequences of miR-497 overexpression were evaluated in proliferation and colony-formation assays. Concurring with our previous observations, the doxycycline-mediated induction of miR-497 dramatically reduced the proliferation and colony-formation capability of SK-N-BE(2) cells while doxycycline itself did not have any effect (Figure 4A–4C). These results were confirmed in the MYCN-amplified cell line LA1-5s (Supplementary Figure 3).

Next, we established NB tumors by the subcutaneous injection of 5 × 10^6^ SK-N-BE(2) cells previously transduced with the pTRIPZ-miR-497 vector, into the right flank of NMRI-nude mice (Figure 4D). Once tumors were detected (day 4–5 post-injection), mice were randomized into two groups; while the control group received vehicle (2% sucrose), the miR-497 group received 1mg/ml doxycycline and 2% sucrose in drinking water *ad libitum*. Tumor volume was monitored every 2–3 days over a three-week period. The overexpression of miR-497 in the presence of doxycycline showed a ∼2–3 fold reduction in tumor growth (day 20–22) (Figure 4E) and tumor weight (Figure 4F) compared to doxycycline-untreated mice. PCR analysis confirmed the activation of miR-497 expression by doxycycline (Supplementary Figure 2C).

**Figure 5:**
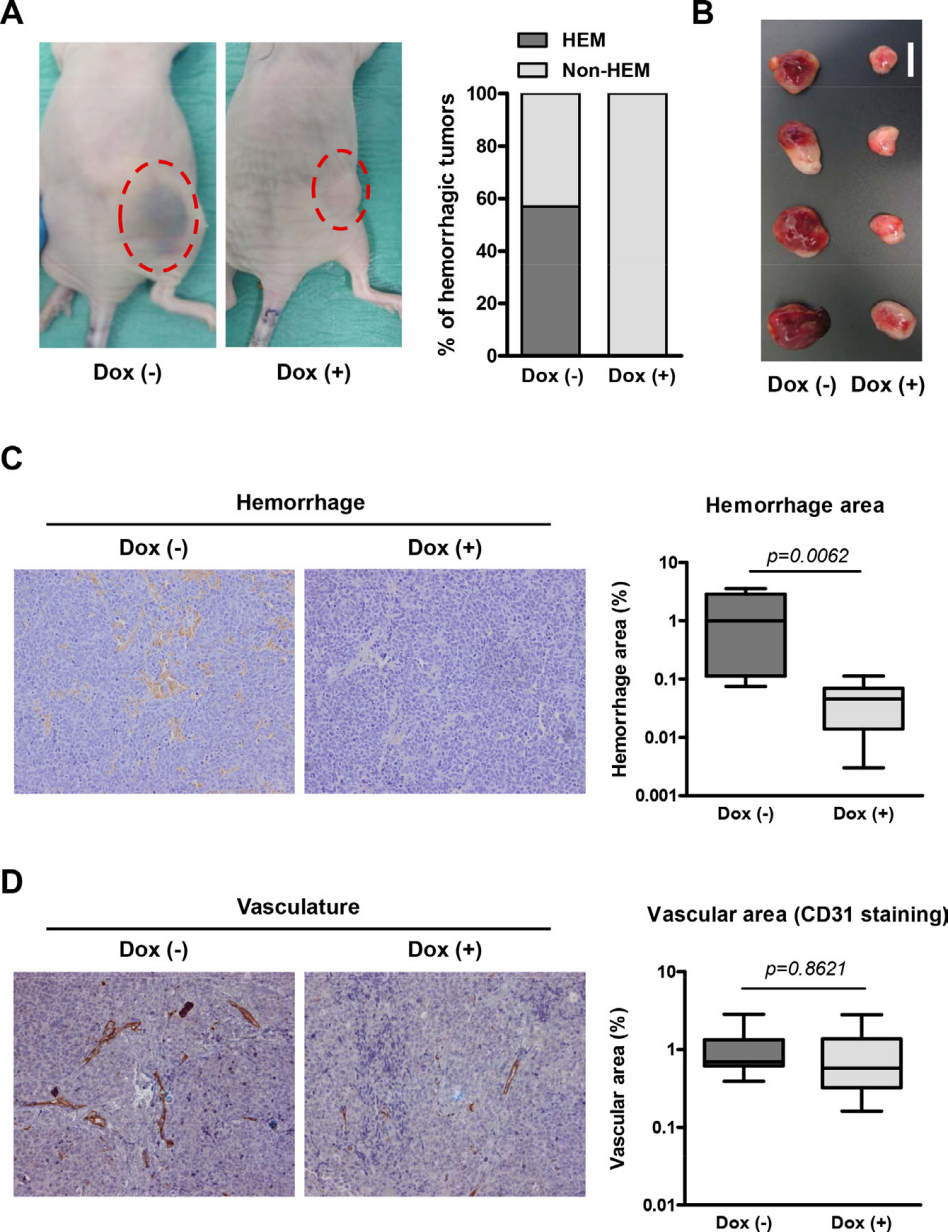
Overexpression of miR-497 *in vivo* reduces permeability of blood vessels (**A**) Representative images of mice bearing NB xenografts with and without doxycycline treatment and quantification of mice with hemorrhagic tumors. (**B**) Representative images of dissected tumors (bar indicates 1 cm). (**C**) Hemorrhagic area was determined developing endogenous tissue peroxidase (mostly from red blood cells) using DAB precipitate deposition. Representative histology images of dissected tumors and quantification of hemorrhage areas of the images. (**D**) Vascularization was determined using an anti-CD31 (PECAM1) antibody and Envision anti-Rabbit HRP-conjugated secondary antibody complex. Representative histology images of dissected tumors and quantification of vascular areas of the images. Quantification of both vascularization and hemorrhaging was made from 3 representative images of 4 independent tumors per group.

During the course of the experiment, the majority of mice bearing NB xenografts without doxycycline displayed hemorrhagic tumors whereas none of the tumors in the doxycycline-treated arm did so (Figure 5A), possibly indicating specific effects of miR-497 expression in the tumor vasculature. This effect was further visible when tumors were dissected (Figure 5B). Histologic analysis revealed extensive hemorrhagic areas in the control group (Figure 5C) and few red blood cells in the parenchyma of miR-497-overexpressing tumors. The presence of red blood cells not exclusively inside or in the proximity of blood vessels results from high permeability of tumor blood vessels, a common trait of human NB. Additionally, the number and area of blood vessels were measured. Although a trend was observed towards a reduction in blood vessel area in miR-497-overexpressing tumors, differences did not reach statistical significance (Figure 5D).

In summary, the overexpression of miR-497 *in vivo* resulted in reduced tumor growth and less permeability of blood vessels.

### MiR-497 targets several cell cycle, survival and angiogenesis genes

**Figure 6:**
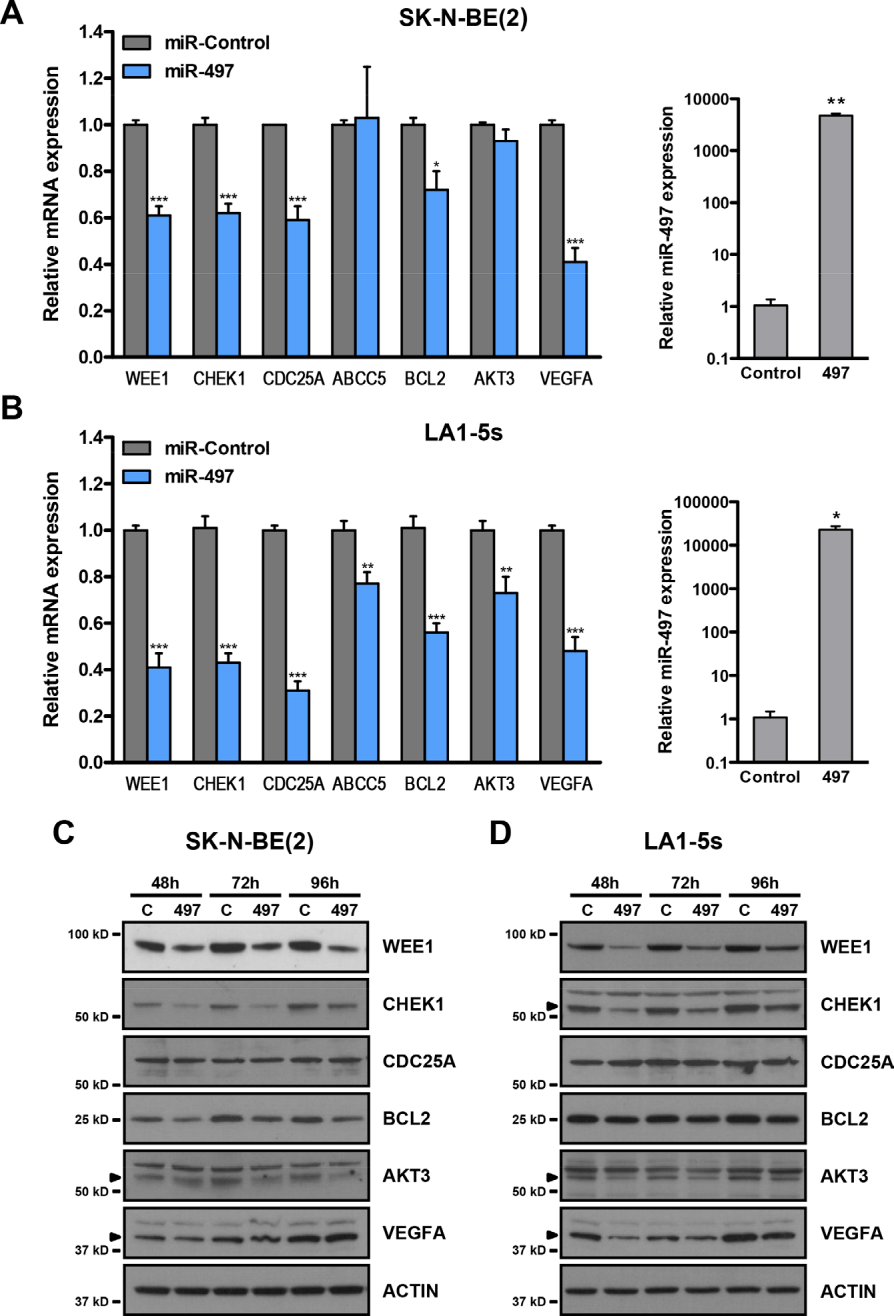
MiR-497 regulates the expression of cell cycle, survival and angiogenesis genes (**A**) SK-N-BE(2) and (**B**) LA1-5s cells were transfected with 25 nM of miR-Control or miR-497 oligonucleotides in triplicate. The expression of the indicated genes (left panels) and miR-497 (right panels) was determined by qRT-PCR at 48 h post-transfection. *p < 0.05; **p < 0.01 and ***p < 0.001. Representative Western blot of the indicated proteins at 48 to 96 h post-transfection in (**C**) SK-N-BE(2) and (**D**) LA1-5s cells. Arrowheads point at the specific band.

The miR-15 family members were initially selected in this study owing to their potential for targeting several chemoresistance-associated genes such as *CHEK1*, *CDC25A*, *WEE1* and *ABCC5*. We proceeded to verify whether these genes were truly modulated by miR-497 overexpression in the neuroblastoma context. We decided to include other potential targets in the analysis that could also be related to the observed phenotype such as *BCL2*, *AKT3* and *VEGFA*. The transient overexpression of miR-497 in SK-N-BE(2) and LA1-5s (MYCN amplified) cell lines (Figure 6A–6B, right panels) proved to cause a reduction in the mRNA levels of *WEE1*, *CHEK1*, *BCL2*, *CDC25A* and *VEGFA* while a reduction in *ABCC5* and *AKT3* was observed only in LA1-5s cells 48 h post-transfection (Figure 6A–6B, left panels). To further confirm whether the reduction in mRNA was followed by a decrease in protein levels, a Western blot was performed at different time-points post-miR-497 transfection. The protein levels of WEE1, CHEK1, BCL2 and AKT3 were reduced as early as 48 h upon miR-497 transfection. VEGFA reduction was much more evident in LA1-5s than in SK-N-BE(2), perhaps due to different protein stability among cell lines. By contrast, the levels of CDC25A remained unaltered in both cell lines (Figure 6C–6D).

**Figure 7:**
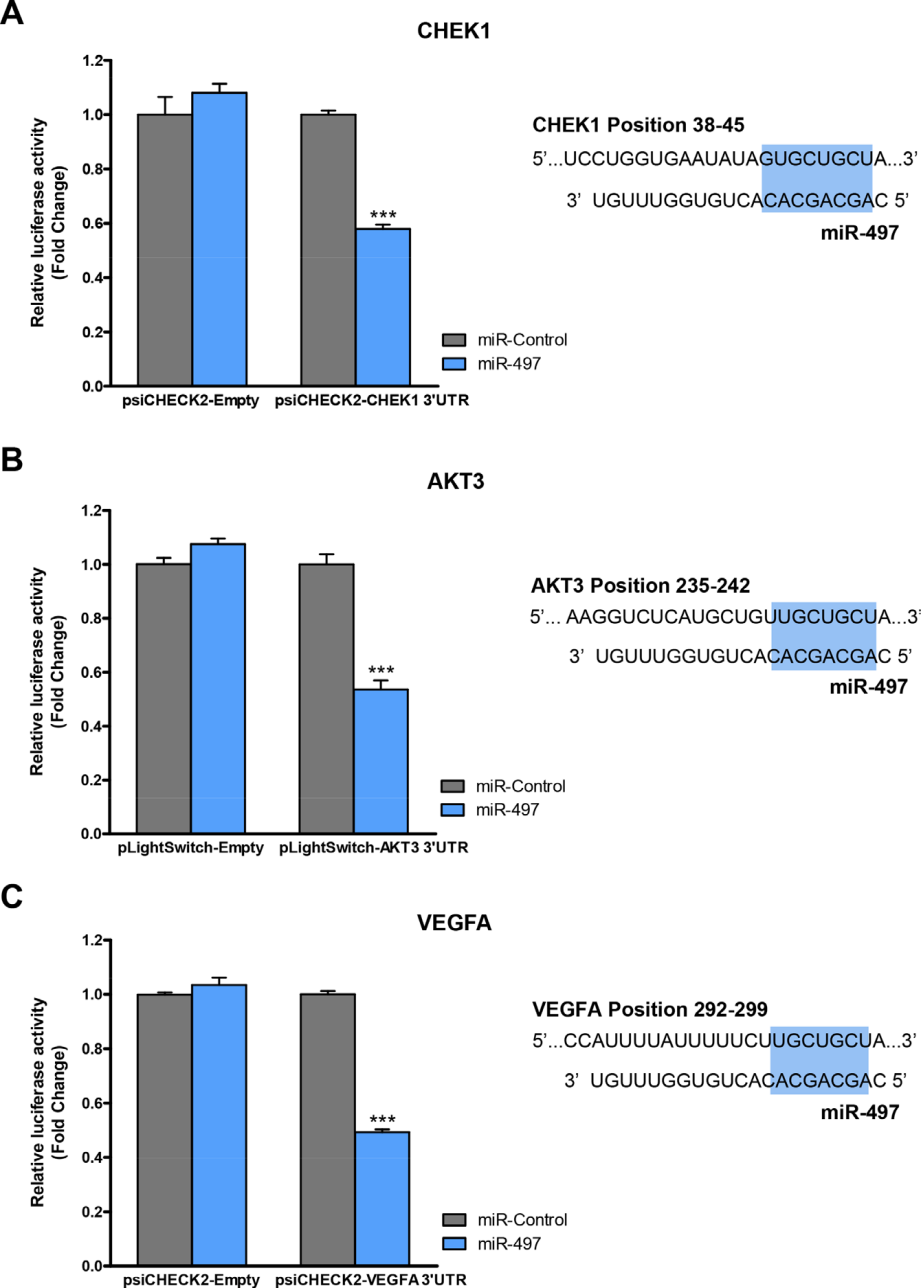
*CHEK1*, *AKT3* and *VEGFA* are miR-497 direct targets HEK-293T cells were co-transfected with 50 ng/well of indicated luciferase-reporter vector and 25 nM of miR-497 or miR-Control. 24 h post-transfection Luciferase Assay for (**A**) *CHEK1*, (**B**) *AKT3* or (**C**) *VEGFA* constructs was performed. Data represent mean ± SEM of three independent experiments (six replicates per experiment). ***p < 0.001. Right panels show the miR-497-binding site sequence within the indicated human genes. Seeding region is highlighted in blue.

Since *WEE1* and *BCL2* have been reported to be direct miR-497 targets in NB cells [28, 29], we proceeded to analyze whether the remaining candidate genes were also directly modulated by miR-497. *CHEK1*-, *AKT3*- and *VEGFA*- 3′UTR luciferase-reporter vectors were engineered and co-transfected with miR-497 or control mimic oligonucleotides (Figure 7). Significant reduction in luciferase activity was observed upon miR-497 transfection for *CHEK1*, *AKT3* and *VEGFA* 3′UTR vectors, thereby indicating a direct modulation of these genes by miR-497.

Collectively, these data suggest that miR-497 may regulate proliferation, survival and tumor vascular permeability by targeting key genes involved in DDR such as *WEE1* and *CHEK1*, cell growth and viability such as *AKT3* and *BCL2* and angiogenesis regulators such as *VEGFA*.

## DISCUSSION

Resistance to current chemo- and radiotherapy treatments remains the main cause of cancer treatment failure. Most of these treatments are aimed at targeting the most-dividing cells interfering in the cell proliferation and division processes, thereby inducing DNA damage and, consequently, activating the DNA-damage response (DDR). The alteration of this process contributes notably to the multi-drug resistance phenotype and consists of a well-orchestrated signaling cascade which halts cell cycle progression and repairs DNA. When DNA damage is beyond repair, cell death mechanisms are triggered. Genetic and/or epigenetic alterations of this complex network, may predispose to cancer, promote tumor progression or participate in the resistance to current therapies [30].

Outcomes for low- and intermediate-risk NB patients are excellent, but survival for high-risk NB is less than 50%. High-risk NB may be initially responsive to DNA-damaging therapies; however, most patients will relapse and be refractory to conventional therapies, rendering NB a paradigm of chemoresistant tumors [31]. The implication of several DDR elements in the resistance of NB to therapy has been proposed, with TP53 [32–34], antiapoptotic Bcl-2 family members such as Bcl-2 [35] or Bcl-XL [36], and checkpoint regulators [37], being the most characterized. Therefore, targeting different DDR components simultaneously may improve the clinical response of high-risk patients.

MiRNAs are small non-coding RNAs that regulate gene expression generally by direct binding to the 3′UTR of their target genes. One of the features that renders miRNAs striking candidates for therapy is that a single miRNA can modulate the expression of multiple genes of the same or different pathways, thus hindering the development of resistance mechanisms by the tumor.

We propose the use of miRNAs as a new clinical tool to treat advanced NB that do not respond to current chemotherapies. We screen the therapeutic potential of miRNAs that could simultaneously target several MDR-related genes that are overexpressed in advanced NB (stage 4 with MYCN amplification). We identified miR-497 as the most anti-proliferative miRNA in two representative NB cell lines resistant to currently-used chemotherapeutic drugs. Low miR-497 expression was associated with shorter event-free survival and MYCN amplification in our cohort of human NB samples, suggesting a tumor-suppressor role. In accordance with our findings, miR-497 has also been found to be underexpressed in advanced stages of cancer or when tumor samples were compared with normal counterpart tissues, and play a tumor-suppressor role in other malignancies such as peritoneal [38], adrenocortical [39], prostate [40] and hepatocellular carcinomas [41, 42]; malignant astrocytoma [43]; neuroblastoma [29]; gastric [44, 45], cervical [46], bladder [47], pancreatic [48], ovarian [49], colorectal [50], non-small cell lung cancer [51], and male [52] and female breast cancer [53–56].

The overexpression of miR-497 reduced the proliferation of all cell lines analyzed and induced apoptosis in MYCN-amplified cell lines. Additionally, we provide the first evidence of antitumoral activity of miR-497 in a preclinical mouse model of pediatric cancer. Concurring with our results, in the majority of cellular models studied, the restoration of miR-497 levels impaired cell cycle progression and/or induced apoptosis *in vitro* and reduced tumor growth *in vivo* [45, 51, 57, 58]. Among miR-497 targets, multiple cell cycle regulators such as CCNE1, CDC25A, CCND3, CDK4 [41] or proteins involved in apoptosis such as BCL2 [44], BCL-w [54] and BIRC5 [47] have been described. In NB, only the WEE1 G2 checkpoint kinase has been recently identified [29].

We observed a downregulation of cell cycle regulators (CHEK1 and WEE1), cell survival proteins (AKT3 and BCL2) and the vascular permeability factor (VEGFA) after miR-497 overexpression and all have been identified as direct miR-497 targets.

One of the targets most affected by the ectopic expression of miR-497 was CHEK1. This cell cycle regulator was also found to be a plausible therapeutic target in NB by RNAi screening of several protein kinases [13]. We found CHEK1 to be highly expressed in advanced NB stages, with higher levels in MYCN-amplified tumors and a clear correlation between high CHEK1 mRNA levels and shorter progression-free and overall survival (Supplementary Figure 4A–4D). An inverse correlation was observed between the mRNA levels of CHEK1 and miR-497 in human NB tumors (Supplementary Figure 4E). Interestingly, simultaneous pharmacologic inhibition of CHEK1 and WEE1, both miR-497 targets, acted synergistically to impair neuroblastoma cell growth *in vitro* and *in vivo* [59], further demonstrating the therapeutic potential of miR-497.

AKT is the central component of the PI3K/AKT/mTOR pathway which is often hyperactivated in NB tumors [60]. This kinase is able to phosphorylate and regulates the function of a plethora of substrates that are critical for growth, proliferation, survival and angiogenesis, among other cellular processes [61]. There are three AKT isoforms which may play different roles in human cancer [62]. Particularly, AKT3 is abundantly expressed in the nervous system and has been associated with the development of neural crest-derived tumors such as melanoma [63] or involved the progression of gliomas through activation of the DNA repair pathway [64]. Interestingly, AKT3 has been shown to modulate angiogenesis through the control of VEGFA expression [65], another of our miR-497-identified targets. Therefore, the downregulation of AKT3 and VEGFA, both of the same axis, may ensure a reduction in the production and secretion of VEGFA. In our established xenografts, the inducible expression of miR-497 was able to reduce tumor growth and the permeability of blood vessels. This latter effect could be particularly relevant for cancer therapy. A disorganized and leaky blood vessel network is frequently found in solid tumors and drives tumor-induced angiogenesis, blood flow disturbances, inflammatory cell infiltration, and tumor cell extravasation. Moreover, it can limit the efficacy of conventional therapies by impairing intravenous drug delivery. It is now accepted that not only excessive angiogenesis should be controlled but also vascular hyperpermeability [66]. High vascularization is associated with poor prognosis in NB [67] and combined treatments with anti-angiogenic agents showed promising benefits in preclinical models. Bevacizumab is a humanized monoclonal anti-VEGF antibody that neutralizes all isoforms of human VEGF. Dickson et al. reported that bevacizumab-mediated VEGF blockade caused alterations in tumor vessel physiology that permitted improved delivery and efficacy of chemotherapy [68, 69].

The use of miRNAs as therapeutic tools has recently entered in clinical development. One of the major challenges for miRNA-based therapies is to improve miRNA delivery, bioavailability and specific targeting to tumoral cells. In the field of oncology, the first molecule to enter in a phase I clinical trial was MIRX34 [70]. This compound is a double-stranded RNA mimic of the tumor suppressor microRNA, miR-34, encapsulated in a liposomal nanoparticle formulation. A limitation of this formulation is that when administered intravenously, its distribution is concentrated mainly in the liver, where it is very effective, but is metabolized and excreted quickly, limiting its therapeutic potential in less irrigated tissues [71]. In order to improve the specific targeting to tumoral cells, a recently-open phase I clinical trial sponsored by the University of Sidney, propose the use of miR-15/16 mimics packaged in EDV^™^ nanocells targeted with Epidermal Growth Factor Receptor antibodies (TargomiRs) for the treatment of patients with recurrent malignant pleural mesothelioma and non-small cell lung cancer [72].

The downregulation of multiple DDR-related targets by a single miRNA, make miR-497 as a good therapeutic candidate not only as a monotherapy, but also in combination with current treatments. For example, miR-497 overexpression was reported to increase cisplatin-induced apoptosis in NB cell lines [29] or reduce the resistance to cisplatin in ovarian cancer using *in vitro* and *in vivo* models [58]. In line with the same evidence, high levels of miR-497 were found to be associated with better prognosis due to increased chemosensitivity in diffuse large B-cell lymphoma (DLBCL) patients. In this study, ectopic expression of miR-497 in DLBCL cell lines led to a reduction on cell viability after exposure to rituximab and different chemotherapeutics relevant in multi-agent lymphoma therapy [73].

In summary, our findings unveil miR-497 as a tumor-suppressor molecule in NB. The restoration of miR-497 may represent a novel and feasible therapeutic approach to treat high-risk NB by downregulating the expression of key proteins involved in DDR such as the cell cycle regulators WEE1 and CHEK1, cell growth and survival, AKT3 and BCL2 and the vascular permeability regulator VEGFA.

## MATERIALS AND METHODS

### Cell lines

CHLA-90 cell line was obtained from the Children's Oncology Group cell line repository. SK-N-BE(2), LA1-5s and SK-N-AS cell lines were purchased from ATCC. All cell lines obtained directly from the tissue banks were amplified and stored in liquid nitrogen. Upon resuscitation, cells were maintained in culture for no more than 2 months. All cell lines were cultured and maintained in Iscove's Modified Dulbecco's Medium (Life Technologies, Thermo Fisher Scientific), supplemented with 20% heat-inactivated FBS (South America Premium, Biowest), 1% Insulin-Transferrin-Selenium G Supplement (Life Technologies, Thermo Fisher Scientific), 100 U/ml penicillin and 100 μg/mL streptomycin (Life Technologies, Thermo Fisher Scientific) and 5 μg/ml of Plasmocin Prophylactic (InvivoGen). All cultures were maintained at 37°C in a saturated atmosphere of 95% air and 5% CO_2_.

### Cell proliferation (crystal violet)

Liposomal transfection complexes without miRNA (MOCK) or with miRIDIAN microRNA Mimic oligonucleotides, Negative Control or Transfection Control with Dy547 (Dharmacon, GE Healthcare, 25 nM per well) were generated with Lipofectamine 2000 (Life technologies, Thermo Fisher Scientific, 0.2 μL per well) in 96-well plates (6 replicates/condition) following the manufacturer's recommendations. Cells were seeded at specific densities (SK-N-BE(2) 10 × 10^3^ cells/well, LA1-5s 5 × 10^3^ cells/well, SK-N-AS 8 × 10^3^ cells/well, CHLA-90 16 × 10^3^ cells/well) into wells containing liposomal complexes followed by overnight incubation in a humidified incubator at 37°C and 5% CO_2_. Media were changed 12–16 h later. At the indicated time points, cells were fixed with 1% glutaraldehyde (Sigma-Aldrich) in PBS for 20 min and stored in PBS at 4°C. At the end of the experiment, cells were stained with 0.5% crystal violet (Sigma-Aldrich) for 20 min followed by extensive washing with diH_2_O. Crystals were dissolved with 15% acetic acid (Fisher Scientific) and optical density was read at 590 nm using an Epoch Microplate Spectrophotometer (Biotek).

### Analysis of miRNA expression in human samples

Gene and miRNA expression data from neuroblastoma tumors was obtained from the Tumour Neuroblastoma Compendium (NRC) dataset. A total of 365 neuroblastoma samples were obtained and analyzed from patients enrolled by Our Lady's Hospital for Sick Children (Crumlin, Dublin, Ireland), the Children's Oncology Group (Philadelphia, USA), the Ghent University Hospital (Ghent, Belgium), the Academic Medical Center (AMC; Amsterdam, Netherlands) and the University Children's Hospital Essen (Essen, Germany). This work was approved by the Research Ethics Committee of each participating institute. Informed consent was obtained from the patients' relatives. The investigators who deposited the data in the R2 repository agree to share the data for this work. Patient characteristics are listed in Supplementary Table 4. Tumors were profiled for 430 miRNAs plus 36 control small RNAs using individual Taqman PCR assays setup in 384-well format and mRNA gene expression profile was performed by Affymetrix GeneChip HG-U133plus2.0. The data set has been previously described in a number of publications [74–78]. Kaplan-Meier analysis was carried out using the scan function of the R2 data analysis tool at the University of Amsterdam (http://R2.amc.nl). The R2 Kaplan-Meier scanner for a single miRNA split the group in two and produced a graph containing 2 *p*-values. A raw *p*-value represented the log rank statistical significance. A Bonferroni corrected *p*-value indicated the significance in survival potential.

### Cell death and caspase activity

CHLA-90, SK-N-AS, LA1-5s and SK-N-BE(2) cells were seeded at 105 × 10^3^, 54 × 10^3^, 35 × 10^3^ and 67 × 10^3^ cells/24 well-plates, respectively. Cells were reverse transfected using Lipofectamine 2000 with miRIDIAN microRNA Mimic Negative Control and miRIDIAN microRNA Mimic miR-497 (25 nM) in triplicate and 6–8 h later media were changed. 96 h post-transfection, cell death was analyzed by Hoechst staining. Apoptosis quantification was made from 4 representative images/well (*n* = 3 replicates per condition). Cells with uniformly stained chromatin were scored as healthy whereas those with fragmented and/or condensed chromatin were scored as apoptotic.

For caspase-3/7 activity assays, SK-N-BE(2) and LA1-5s cells were seeded in 60-cm dishes and reverse transfected with 25 nM of Negative Control or miR-497 oligonucleotides in triplicate. At 72 h post-transfection, cells were harvested and incubated for 15 minutes on ice with caspase-activity buffer (20 mM HEPES/NaOH pH 7.2, 10% sucrose, 150 mM NaCl, 5 mM EDTA, 1% Nonidet P-40, 0.1% CHAPS and 1x EDTA-free complete protease inhibitor cocktail (Roche)). Lysates were cleared by centrifugation at 13,000 × g for 5 min at 4°C, and supernatants were quantified by the Lowry method (Bio-Rad). Assays were performed in triplicate using 15–25 μg of protein in the same lysis buffer supplemented with 10 mM DTT and 25 μM of the fluorogenic caspase-3 substrate Ac-DEVD-AFC (Calbiochem). Plates were read in an Appliskan (Thermo Fisher Scientific) microplate reader using a 405 nm excitation filter and a 535 nm emission filter.

### Plasmids, lentivirus production and transduction

pTRIPZ was purchased from Open Biosystems (Dharmacon, GE Healthcare). To generate the miR-497 expression vector, a fragment of 184 bp of pre-miR-497 was amplified by PCR (Fw: 5′- GTTAACCTTCCCAGCACTGCTATGTG-3′ and Rv: 5′-CTCGAGTGTCAACTTCTCCAGGATGG-3′) from genomic DNA and cloned directly into the pTRIPZ-empty vector using HpaI/XhoI restriction sites. Lentiviruses were propagated using previously described methods [79, 80]. SK-N-BE(2) and LA1-5s cells were seeded in 100 mm-dishes and incubated overnight prior to infection. Medium was replaced with viral supernatant supplemented with 5 μg/mL polybrene, and incubated for 24 h, followed by replacement with growth medium. pTRIPZ-Control or pTRIPZ-miR-497 stably-transduced SK-N-BE(2) and LA1-5s cells were selected with 1 μg/mL puromycin (Sigma-Aldrich) prior to use in experiments.

### Colony formation assay

pTRIPZ-Control or pTRIPZ-miR-497 stable SK-N-BE(2) cells were seeded at 10,000 cells/well and pTRIPZ-Control or pTRIPZ-miR-497 stable LA1-5s cells were seeded at 1,000 cells/well in 6-well plates in triplicates with 0.5 μg/mL of puromycin and with or without doxycycline (0.5–1 μg/mL). Medium was refreshed every 3 days and cells were allowed to grow for 10–15 days. Then, cells were stained with crystal violet, photographed and scored

### Cell proliferation assay (cell counting)

pTRIPZ-Control or pTRIPZ-miR-497 stable SK-N-BE(2) cells were seeded at 250,000 cells and pTRIPZ-Control or pTRIPZ-miR-497 stable LA1-5s cells were seeded at 50,000 cells into 35 mm-dish with 0.5 μg/mL of puromycin and with or without doxycycline (0.5–1 μg/mL). Cells were counted and reseeded at days 3, 6 and 9 (*n* = 3/condition).

### Mouse xenograft

SK-N-BE(2) cells transduced with pTRIPZ-miR-497 (*n* = 20) were injected (5 × 10^6^ cells/flank) into the right flank of 6–8-week-old female NMRI-nude mice (Janvier Labs, Le Genest-Saint-Isle, France). Cells were injected in 300 μl of PBS:Matrigel (Corning) (1:1). Once tumors were detected (day 4–5 post-injection), mice were randomized into two groups (*n* = 10/group). Doxycycline (1 mg/mL) was added (*n* = 10, miR-497 group) or not (*n* = 10, control) to the drinking water with 2% sucrose (Sigma-Aldrich). Tumor volume was measured every 2–3 days for three weeks. At the end of the experiment, the primary tumors were excised and weighed. Tissues were fixed in 10% formalin, and paraffin-embedded.

### Immunohistochemistry

Vascularization was determined by a standard immunohistochemistry method in tissue sections using an anti-CD31 (PECAM1) antibody (ab28364, Abcam) and Envision anti-Rabbit HRP-conjugated secondary antibody complex (Dako). Hemorrhaging was determined by developing endogenous tissue peroxidase (mostly from red blood cells) using DAB precipitate deposition (Dako). Quantification of both vascularization and hemorrhaging was made from 3 representative images of 4 independent tumors per group, by determining area density (area positive for marker by total viable tumor area) using Image J software (NIH USA, open-source, public domain software).

### Gene expression analysis by quantitative real-time PCR

Total RNA, including the small RNA fraction, was extracted using the miRNeasy Mini Kit (Qiagen). mRNAs were reverse transcribed (0.5–1 μg total RNA) using Taqman RT kit (Applied Biosystems, Thermo Fisher Scientific), and mature miRNA expression analysis was quantified using Taqman microRNA assays (Applied Biosystems, Thermo Fisher Scientific) following manufacturer's recommendations. cDNA was quantified by standard RT-qPCR methodology using 2X Power SYBR Green Master Mix (Applied Biosystems, Thermo Fisher Scientific). Gene expression was normalized against the *L27* housekeeping gene for mRNA, and U6 or RNU-44 small RNA for miRNA analysis. The primer sequences are listed in Supplementary Table 5. The relative fold-change in expression was determined by the comparative 2^(−ΔΔCT)^ method [81].

### Western blot

Cell homogenates were obtained in RIPA buffer (Pierce, Thermo Scientific), supplemented with 1 × EDTA-free complete protease inhibitor cocktail (Roche). Protein concentration was quantified by a modified Lowry assay (DC protein assay; Bio-Rad). 30 μg of protein were resolved in NuPAGE 4-12% Bis-Tris gels and transferred to iBlot Gel Transfer Stacks PVDF membranes (Life Technologies, Thermo Fisher Scientific). After blocking with Tris-buffered saline with Tween-20 containing 5% non-fat dry milk or 5% BSA for 1 h at room temperature, membranes were probed overnight at 4°C with the following antibodies: anti-PARP [1:2000, Cell Signaling #9542]; anti-CDC25A [1:1000, Santa Cruz #sc-7389]; anti-WEE1 [1:1000, Santa Cruz #sc-5285]; anti-CHK1 [1:2000, Santa Cruz #sc-8408]; anti-BCL2 [1:1000, Dako #M0887]; anti-AKT3 [1:1000, Upstate #05-780]; anti-VEGF [1:1000, Abcam #ab46154]; anti-Mouse IgG-Peroxidase antibody produced in rabbit (1:10.000, Sigma-Aldrich #A9044); anti-Rabbit IgG-Peroxidase antibody produced in goat (1:10.000, Sigma-Aldrich #A0545) and anti-Actin HRP [1:40.000, Santa Cruz #sc-1616]. Membranes were developed with SuperSignal Dura detection kit (Pierce) or EZ-ECL Chemiluminescence detection kit (Biological Industries, Kibbutz Beit-Haemek, Israel).

### Luciferase 3′UTR assays

psiCHECK^™^-2 vector was purchased from Promega. psiCHECK2-CHEK1 3′UTR construct was acquired from Addgene (plasmid 29478, [82]). pLightSwitch vector and pLightSwitch-AKT3 3′UTR were kindly provided by Doug Hanniford and Eva Hernando (NYU Langone Medical Center). psiCHECK2-VEGFA 3′UTR vector was generated by amplifying a 512bp fragment of the VEGFA 3′UTR by PCR (Fw: 5′-CTCGAGGAACCAGATCTCTCACCAGGA-3′ and Rv: GCGGCCGCTCTCCTCCTCTTCCCTGTCA-3′) and cloned into the psiCHECK2^™^-2 vector using XhoI and NotI restriction sites.

HEK293T cells were seeded in 96-well plates at 20,000 cells/well in 100 μL/well of complete media without antibiotic. Cells were co-transfected 16–24 h later using Lipofectamine 2000 with 50 ng of psiCHECK2-CHEK1 3′UTR, pLightSwitch-AKT3 3′UTR or psiCHECK2-VEGFA 3′UTR vector and 25 nM of miR-497 mimic or negative control. Liposomal complexes containing 3′UTR and liposomal Mimic miRNAs were prepared separately in 50 μL volume and then added consecutively to 96-well seeded HEK293T cells. Cells were incubated at 37°C and 5% CO_2_ for 20–24 h. Luciferase assay was performed using the Dual-Glo^®^ Luciferase Assay System (Promega) for psiCHECK2 constructs and LightSwitch Luciferase Assay Reagent^™^ (SwitchGear Genomics) for pLightSwitch constructs, respectively, following the manufacturer's recommendations. Luminescence was measured in an Appliskan (Thermo Fisher Scientific) microplate reader. Renilla luciferase activity was normalized to corresponding firefly luciferase activity (pisCHECK2 constructs) and plotted as a percentage of the control.

### Statistical analysis

Unless otherwise indicated, mean values ± SEM are representative of one out of three independent experiments. Statistical significance was determined by two-tailed unpaired Student's *t*-test (GraphPad Prism Software). * means *p* < 0.05, ** means *p* < 0.01 and *** means *p* < 0.001.

## SUPPLEMENTARY MATERIALS FIGURES AND TABLES


