# Supplementary material for: MicroRNA-497 impairs the growth of chemoresistant neuroblastoma cells by targeting cell cycle, survival and vascular permeability genes
Source: Oncotarget. 2016 Jan 25;7(8):9271–87. doi: 10.18632/oncotarget.7005 (PMC4891039; doi:10.18632/oncotarget.7005)
Supplement: Supplementary file 1 [file oncotarget-07-9271-s001.pdf]

MicroRNA-497 impairs the growth of chemoresistant neuroblastoma cells by targeting cell cycle, survival and vascular permeability genes

Supplementary Materials

SUPPLEMENTARY MATERIALS AND METHODS

Analysis of mRNA expression in human neuroblastoma samples

Gene expression data of DDR-associated genes was analyzed in primary neuroblastoma tumors from the publicly available GSE3960 (n = 101) [1] dataset. CHEK1 levels and association with survival analysis were analyzed from the publicly available GSE45547 (n = 643) [2] dataset.

3'UTR Target Analysis

Candidate genes were selected comparing the Stage 4 MYCN-amplified tumors versus the other stages present in the study (i.e. Stage 1, 3 and 4 without MYCN amplification). The 3′untranslated region (UTR) of the candidate genes was scanned through three different miRNA-binding site prediction algorithms: TargetScan [3], PicTar [4] and miRANDA [5]. MiRNAs found to be potential regulators of at least 3 different genes and by, at least, two independent algorithms were selected for functional studies.

Detection of miR-497 in neuroblastoma xenografts

Tumor tissue from NB xenografts was obtained immediately after surgery and snap frozen in liquid nitrogen and stored at −80°C until processing. Total RNA was extracted using the miRNAeasy Mini Kit (Qiagen, Las Matas, Spain). Between 0.5 and 1 µg of total RNA was retrotranscribed using High Capacity RNA-to-cDNA Kit (Applied Biosystems, Alcobendas, Spain). In order to avoid contamination of endogenous miR-497 from mouse tissue (e.g. stromal or blood cells) human-specific primers were designed for miR-497 precursor detection: miR-497_forward, 5′- CTTCCCAGCACTGCTATGTG-3′ and reverse, 5′- TGTCAACTTCTCCAGGATGG-3′. Equal RNA loading was confirmed using the *L27* housekeeping gene: L27–forward: 5′-AGCTGTCATCGTGAAGAA-3′ and L27-reverse, 5′-CTTGGCGATCTTCTTCTTGCC-3′.

REFERENCES

1. Wang Q, Diskin S, Rappaport E, Attiyeh E, Mosse Y, Shue D, Seiser E, Jagannathan J, Shusterman S, Bansal M, Khazi D, Winter C, Okawa E, et al. Integrative genomics identifies distinct molecular classes of neuroblastoma and shows that multiple genes are targeted by regional alterations in DNA copy number. Cancer Res. 2006; 66:6050–6062.
2. Kocak H, Ackermann S, Hero B, Kahlert Y, Oberthuer A, Juraeva D, Roels F, Theissen J, Westermann F, Deubzer H, Ehemann V, Brors B, Odenthal M, et al. Hox-C9 activates the intrinsic pathway of apoptosis and is associated with spontaneous regression in neuroblastoma. Cell Death Dis. 2013; 4:e586.
3. Grimson A, Farh KK, Johnston WK, Garrett-Engele P, Lim LP, Bartel DP. MicroRNA targeting specificity in mammals: determinants beyond seed pairing. Mol Cell. 2007; 27:91–105.
4. Krek A, Grun D, Poy MN, Wolf R, Rosenberg L, Epstein EJ, MacMenamin P, da Piedade I, Gunsalus KC, Stoffel M, Rajewsky N. Combinatorial microRNA target predictions. Nat Genet. 2005; 37:495–500.
5. Betel D, Wilson M, Gabow A, Marks DS, Sander C. The microRNA.org resource: targets and expression. Nucleic Acids Res. 2008; 36:D149–153.

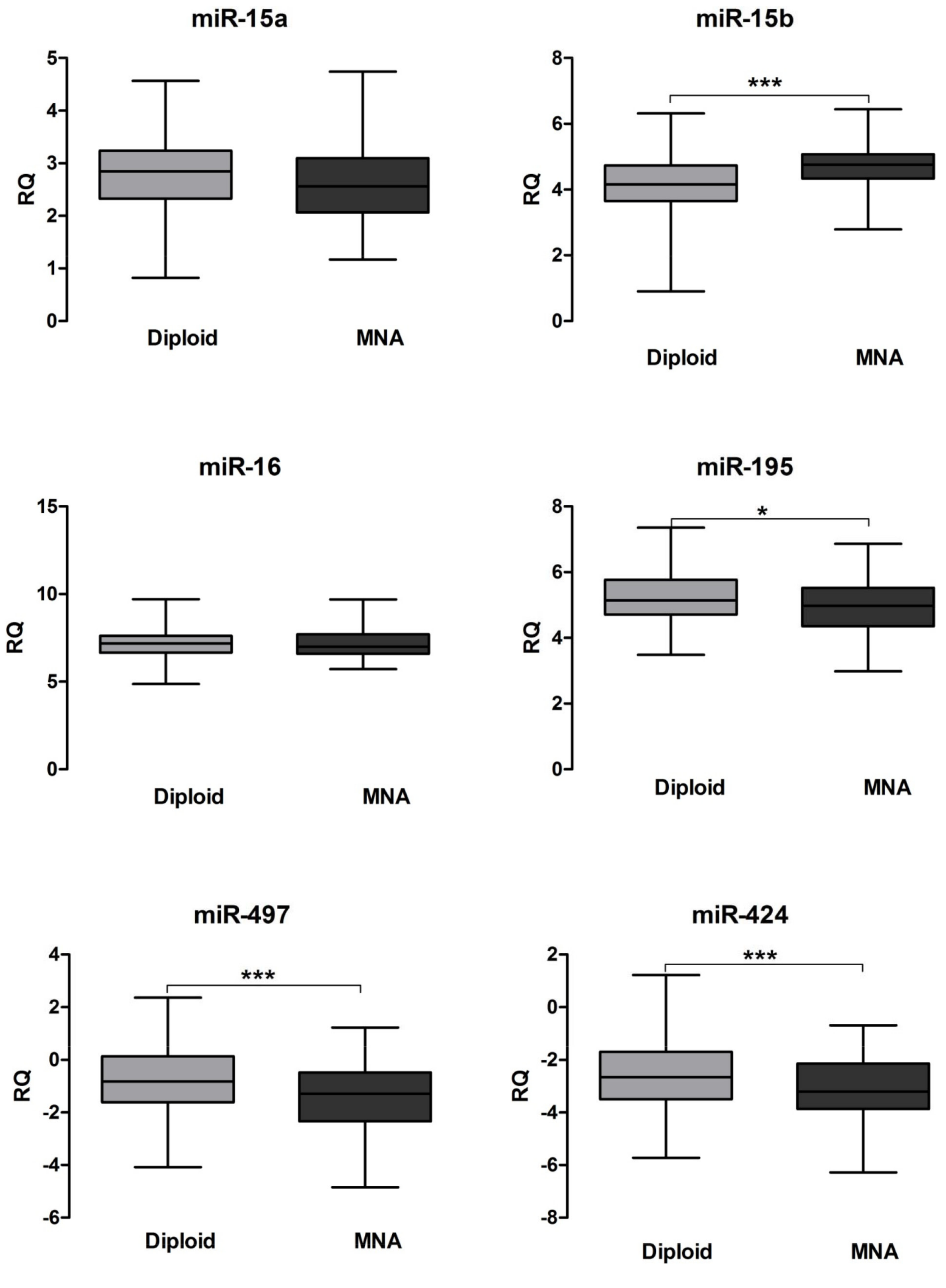

Supplementary Figure S1: MiR-195, miR-497 and miR-424 showed reduced expression levels in MYCN-amplified tumors. Box plots represent mir-15 family member's expression values comparing normal MYCN content (Diploid, n = 265) versus MYCN amplified (MNA, n = 65) tumors. RQ: Relative quantification. *$p < 0.05$; ***$p < 0.001$.

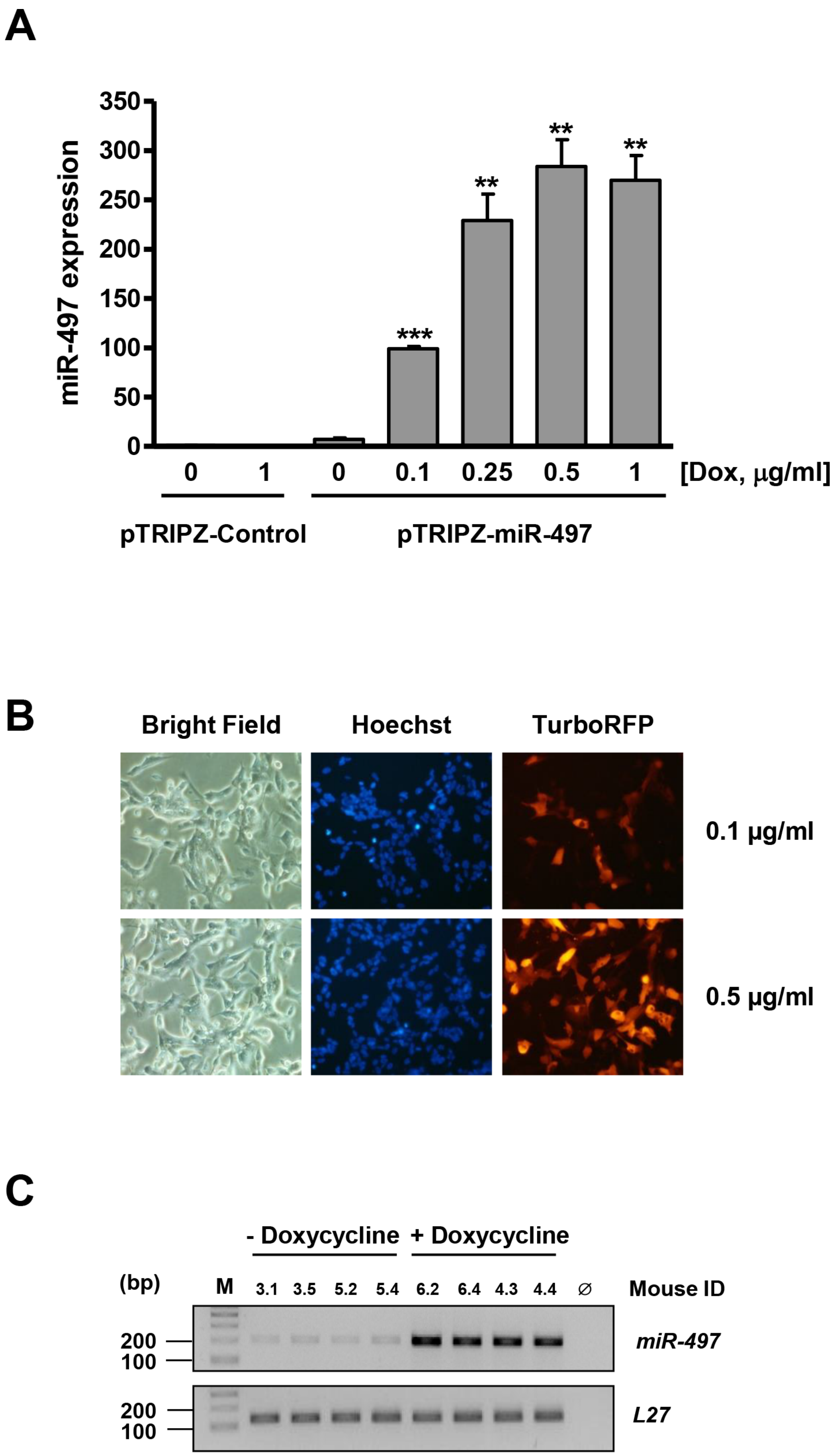

Supplementary Figure S2: Inducible expression vector for miR-497. (**A**) pTRIPZ-Control or pTRIPZ-miR-497-stably transduced SK-N-BE(2) cells were treated with the indicated doses of doxycycline for 72 h. MiR-497 expression was quantified by qPCR. (**B**) Representative images of pTRIPZ-miR-497-SK-N-BE(2) infected cells treated with 0.1 and 0.5 µg/mL of doxycycline for 72 h. TurboRFP (Red Fluorescence Protein) and miR-497 are part of a single transcript allowing the visual marking of miR-497 expressing cells. (**C**) RT-PCR of pre-miR-497 in SK-N-BE(2) xenografts, showing effective miR-497 overexpression in doxycycline-treated mice.

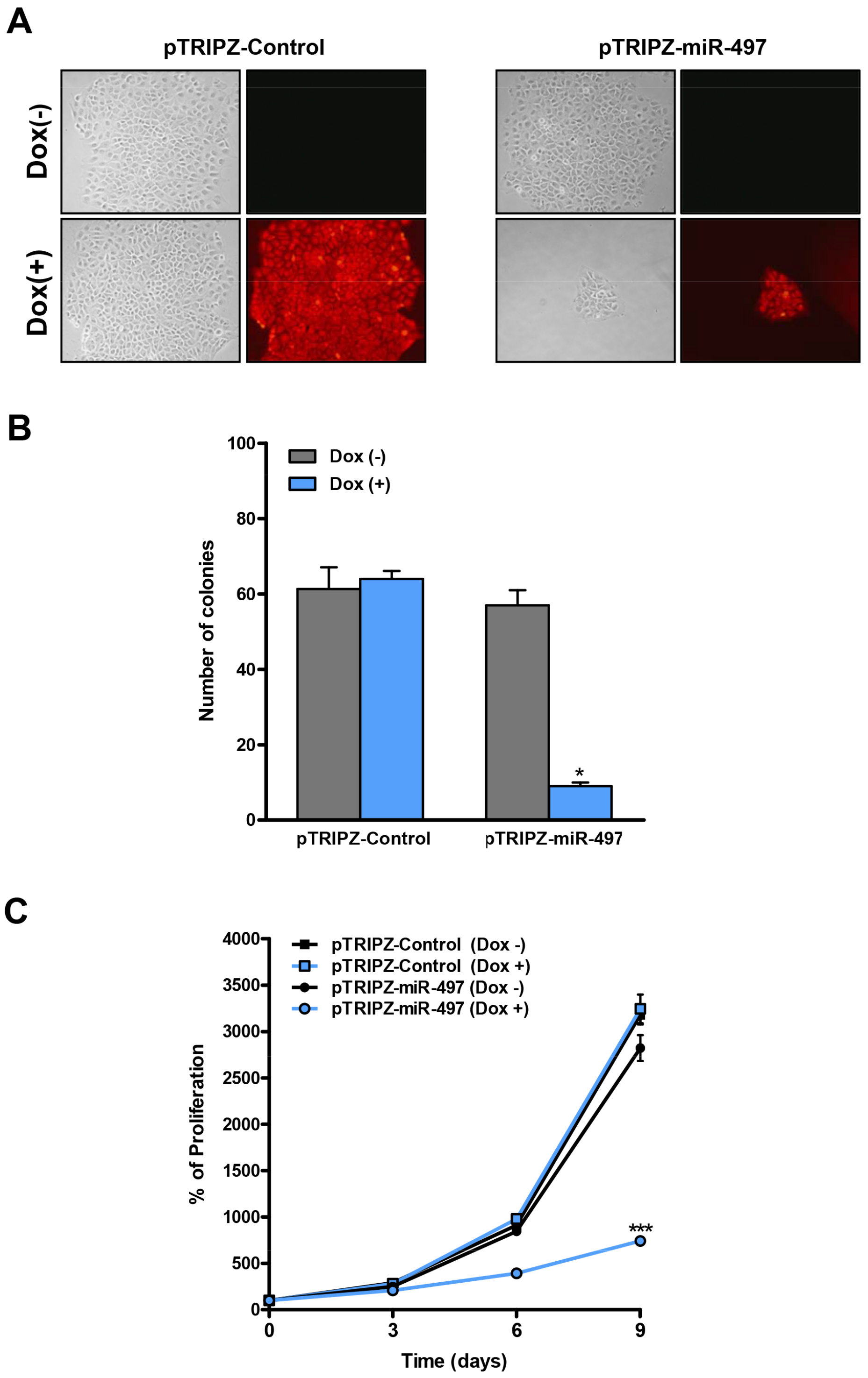

Supplementary Figure S3: Inducible expression of miR-497 impairs colony formation and proliferation in LA1-5s cell line. (**A**) Representative images of a colony-formation assay in pTRIPZ-Control or pTRIPZ-miR-497 stably-transduced LA1-5s cells treated with or without doxycycline (1 µg/mL) and allowed to grow for 9 days (n = 3). The expression of TurboRFP allows the visual marking of miRNA expressing cells. (**B**) Colonies were stained with crystal violet, photographed and the number of colonies was quantified. (**C**) Proliferation time course comparing pTRIPZ-Control or pTRIPZ-miR-497 stably-transduced LA1-5s cells with or without 1 µg/mL of doxycycline. Data represent mean ± SEM of three independent experiments (3 replicates per condition for each experiment). * or *** indicated statistically significant difference comparing cells treated with or without doxycycline at $p < 0.05$ or $p < 0.001$, respectively.

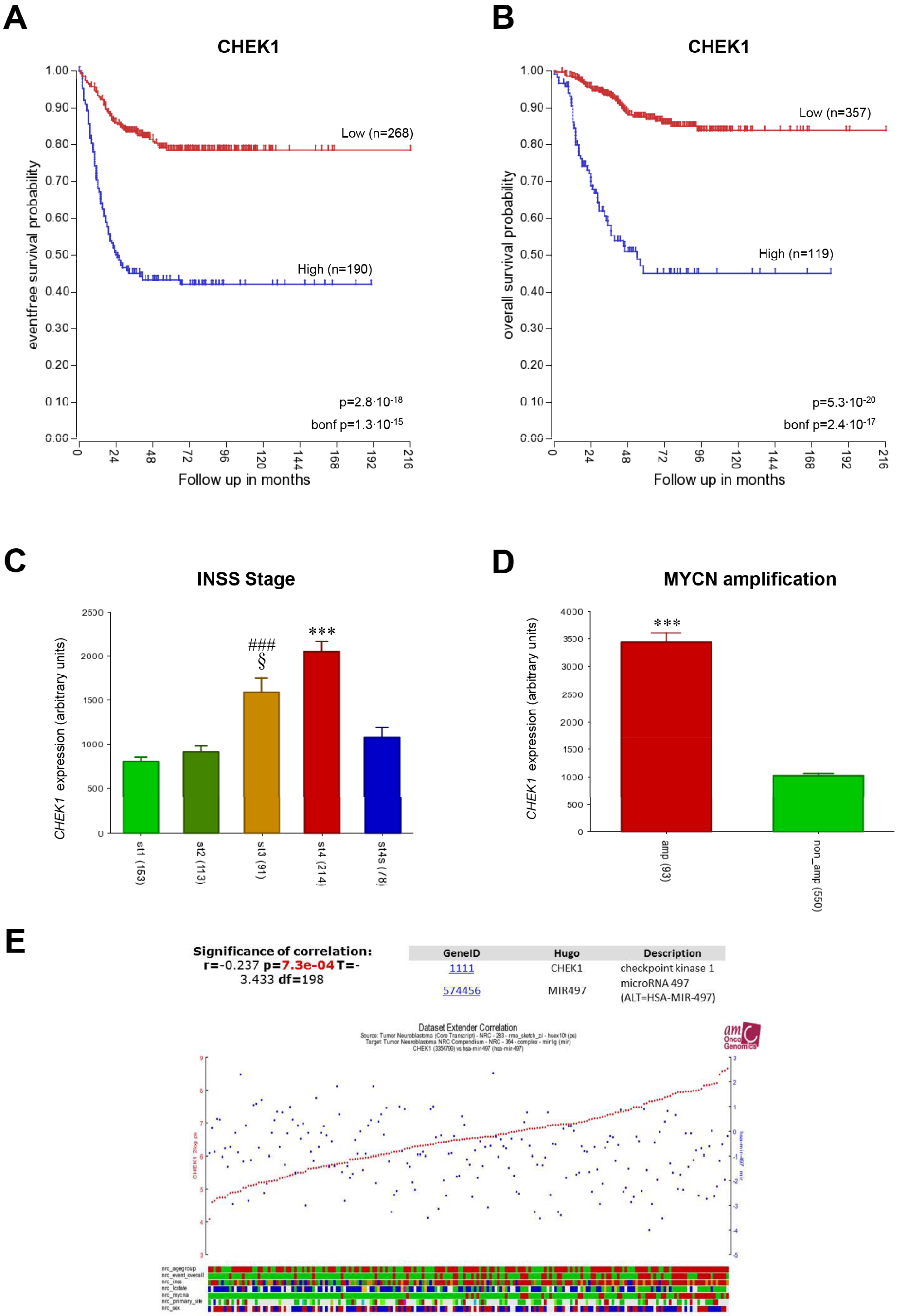

Supplementary Figure S4: *CHEK1* is highly expressed in advanced neuroblastoma and inversely correlates with miR-497. Kaplan-Meier progression-free (**A**) and overall (**B**) survival analysis of *CHEK1* mRNA in human NB tissues (n = 476). (**C**) *CHEK1* mRNA expression according to the International Neuroblastoma Staging System (INSS). *** means $p < 0.001$ for stage 4 versus stage 1, 2 and 4S. ### means $p < 0.001$ for stage 3 versus stage 1. § means $p < 0.05$ for stage 3 versus stage 2. (**D**) *CHEK1* mRNA expression comparing NB with genomic amplification of MYCN versus non-amplified (n = 643). *** means $p < 0.001$. (**E**) Expression correlation between CHEK1 mRNA and miR-497 in human NB tissue samples.

Supplementary Table 1: DNA Damage Response/Detox Pump genes overexpressed in Stage 4 MYCN amplified NB samples

	Gene Symbol	Fold Change (*)	*P* Value
**SENSORS**	H2AFX	2.43	$3.9 \cdot 10^{-05}$
	RNF8	4.33	$8.6 \cdot 10^{-05}$
**TRANSDUCERS**	ATM	1.4	$6.8 \cdot 10^{-03}$
	ATR	1.38	$2.0 \cdot 10^{-04}$
**EFFECTORS**	XRC66	1.33	$3.3 \cdot 10^{-04}$
	PRKDC	1.53	$2.4 \cdot 10^{-05}$
	ATRX	1.34	$3.0 \cdot 10^{-03}$
	CHEK1	1.64	$1.5 \cdot 10^{-04}$
	CHEK2	2.04	$4.2 \cdot 10^{-04}$
	CDK2	1.44	$1.4 \cdot 10^{-02}$
	CDC25A	5.68	$2.7 \cdot 10^{-03}$
	SMC1A	1.73	$3.5 \cdot 10^{-05}$
	CCNB1	2.49	$2.0 \cdot 10^{-04}$
	CDK1	1.94	$1.8 \cdot 10^{-03}$
	PLK1	1.89	$4.4 \cdot 10^{-05}$
	WEE1	1.33	$1.3 \cdot 10^{-02}$
**DETOX/PUMPS**	CES2	1.51	$5.9 \cdot 10^{-04}$
	ABCC1	1.96	$2.1 \cdot 10^{-06}$
	ABCC4	2.36	$2.5 \cdot 10^{-04}$
	ABCC5	1.42	$3.8 \cdot 10^{-02}$
	UMPS	1.70	$1.1 \cdot 10^{-04}$
	RRM1	1.87	$1.1 \cdot 10^{-05}$
	ABCB7	1.56	$6.7 \cdot 10^{-05}$

(*) GSE-3960 Stage 4 MYCN amplified versus rest of stages (1, 3 and 4 MYCN non-amplified)

Supplementary Table 2: List of microRNAs that potentially regulate 3 or more upregulated genes listed in Table 1

microRNA	miR-Family	Target Genes (*)	# Targets
miR-93	miR-17	CDK2, CDC25A, CCNB1, WEE1, ABCC5, CES2	7
miR-7		ATRX, CHEK1, SMC1A, CDK1, ABCC1, ABCC5	7
miR-300	miR-154	ATR, ATRX, CDC25A, CDK1, CCNB1, ABCC4, ABCC5	7
miR-186		CDK2, CDK1, ABCC1, ABCC5, UMPS	6
miR-101		ATRX, CCNB1, ABCC1, ABCC5, RRM1	6
let-7e	let-7	RNF8, CDC25A, SMC1A, ABCC1, ABCC5	5
miR-379		CCNB1, CDK1, XRCC6, ABCC1	5

miR-490-3p		CDK2, CDK1 ABCC4, ABCC5	5
miR-421	miR-95	ATM, CHEK1, ABCC4, ABCC5	4
miR-410	miR-154	CDK2, CDK1, CCNB1, ABCC5	4
miR-340-5p		CDK2, CDK1, CCNB1, ABCC5	4
miR-23a	miR-23	PLK1, ABCC4, ABCC5, UMPS	4
miR-200b	miR-200	CDK2, CDK1, CDC25A, ABCC1	4
miR-497	miR-15	CHEK1, CDC25A, WEE1, ABCC5	4
miR-24		H2AFX, RNF8, CDC25A, CES2	4
miR-146a	miR-146	ATR, CDK1, ABCC5, UMPS	4
miR-320a	miR-320	CHEK1, ATRX, ABCC5, UMPS	4
miR-125a-3p	miR-125	CES2, ABCC4, ABCC5	4
miR-590-3p		CCNB1, CDK1, ABCC5	4
miR-543	miR-329	CDK1, CES2, ABCC4	3
miR-505		CHEK1, ABCC4, ABCC5	3
miR-34a	miR-34	CDC25A, ABCC1, ABCC5	3
miR-339-5p		CDK2, CDC25A, RRM1	3
miR-335		CDK2, CDK1, ABCC5	3
miR-185		ABCC1, ABCC5, UMPS	3
miR-140-5p		CDK1, CCNB1, ABCC4	3
miR-129		CDK1, CES2, ABCC5	3
miR-181b	miR-181	RNF8, CDK1, CCNB1	3

(*) Target analysis predicted, at least, by two independent algorithms

Supplementary Table 3: miR-15 family members

miRNA	Accession	Sequence	Locus	Clustered miRNAs
miR-15a	MIMAT0000068	UAGCAGCACAUAAUGGUUUGUG	13q14.2	miR-16-1
miR-15b	MIMAT0000417	UAGCAGCACAUCAUGGUUUACA	3q25.33	miR-16-2
miR-16-1	MIMAT0000069	UAGCAGCACGUAAAUAUUGGCG	13q14.2	miR-15a
miR-16-2	MIMAT0000069	UAGCAGCACGUAAAUAUUGGCG	3q25.33	miR-15b
miR-195	MIMAT0000461	UAGCAGCACAGAAAUAUUGGC	17p13.1	miR-497
miR-497	MIMAT0002820	CAGCAGCACACUGUGGUUUGU	17p13.1	miR-195
miR-424	MIMAT0001341	CAGCAGCAAUUCAUGUUUUGAA	Xq26.2	miR-503 miR-542 miR-450a-2 miR-450a-1 miR-450b

Supplementary Table 4: Patient characteristics

Variable	*n* (%)
INSS Stage	
1	41 (11.23)
2	100 (27.39)
3	53 (14.52)
4	142 (38.90)
4s	29 (7.94)
MYCN status*	
Amplified	65 (19.69)
Non-amplified	265 (80.31)
Chromosomal imbalance*	
−11q	28 (26.92)
−1p	51 (36.42)
+17q	126 (54.31)

INSS:International Neuroblastoma Staging System.
*Note that there is no molecular profile for each sample.

Supplementary Table 5: Primers

Gene	Primer sequence (5′ to 3′)
AKT3	Fw: GAACGACCAAAGCCAAACAC
	Rv: TGGGTTGTAGAGGCATCCAT
WEE1	Fw: CACACGCCCAAGAGTTTGC
	Rv: GAGGAGTCTGTCGCACATCA
CHEK1	Fw: GACATTCAGAGGGGCAGGAC
	Rv: GCACCTCGGCGGACTG
CDC25A	Fw: CCCAGCTCGGATGCTTTCC
	Rv: TCACAGGTGACTGGGGTGTA
BCL-2	Fw: TCTTTGAGTTCGGTGGGGTC
	Rv: GTTCCACAAAGGCATCCCAGC
ABCC5	Fw: TGTTGTTAGTGCTGGGCCTC
	Rv: TTGATGAGCTCACCCAGGGAT
L27	Fw: AGCTGTCATCGTGAAGAA
	Rv: CTTGGCGATCTTCTTCTTGCC
VEGFA	Fw: GGGCCTCCGAAACCATGAA
	Rv: GGGACCACTTGGCATGGTG
